# Toward Accurate Indoor Positioning: An RSS-Based Fusion of UWB and Machine-Learning-Enhanced WiFi †

**DOI:** 10.3390/s22093204

**Published:** 2022-04-21

**Authors:** Ghazaleh Kia, Laura Ruotsalainen, Jukka Talvitie

**Affiliations:** 1 Department of Computer Science, University of Helsinki, 00014 Helsinki, Finland; laura.ruotsalainen@helsinki.fi; 2 Unit of Electrical Engineering, Tampere University, 33014 Tampere, Finland; jukka.talvitie@tuni.fi

**Keywords:** fusion, Gaussian process, indoor position estimation, machine learning, RSS, RTT, Two-Way Ranging (TWR), UWB, WiFi

## Abstract

A wide variety of sensors and devices are used in indoor positioning scenarios to improve localization accuracy and overcome harsh radio propagation conditions. The availability of these individual sensors suggests the idea of sensor fusion to achieve a more accurate solution. This work aims to address, with the goal of improving localization accuracy, the fusion of two conventional candidates for indoor positioning scenarios: Ultra Wide Band (UWB) and Wireless Fidelity (WiFi). The proposed method consists of a Machine Learning (ML)-based enhancement of WiFi measurements, environment observation, and sensor fusion. In particular, the proposed algorithm takes advantage of Received Signal Strength (RSS) values to fuse range measurements utilizing a Gaussian Process (GP). The range values are calculated using the WiFi Round Trip Time (RTT) and UWB Two Way Ranging (TWR) methods. To evaluate the performance of the proposed method, trilateration is used for positioning. Furthermore, empirical range measurements are obtained to investigate and validate the proposed approach. The results prove that UWB and WiFi, working together, can compensate for each other’s limitations and, consequently, provide a more accurate position solution.

## 1. Introduction

One of the well-known forms of measurement required in different positioning methods is range measurement. In outdoor environments, range measurement can assist with positioning in scenarios where Global Navigation Satellite System (GNSS) signals are not fully available [1]. They can help in collaborative localization, where one collaborating node is aware of its accurate position, and the other has a low-quality position estimation [2]. In indoor environments, range measurement provides the fundamentals of the multilateration technique that is frequently used in various positioning scenarios [1,3]. Range measurement is mostly conducted using Radio Frequency (RF) signals such as GNSS, radar, WiFi, Bluetooth Low Energy (BLE), UWB, or Light Detection and Ranging (LiDAR)-based methods [4]. There are different approaches to finding the range between a transmitter and a receiver using RF signals. Two main methods include using the Received Signal Strength (RSS) values [5,6] and the Time-of-Flight (ToF) of the signals. The use of standalone RSS-based methods is vulnerable to variations in range measurement due to path loss model errors [7]. ToF-based methods include Time of Arrival (ToA), Time Difference of Arrival (TDoA), Two-Way Ranging (TWR), and Round Trip Time (RTT). ToA is the most well-known ranging technique. It depends on knowing the accurate time of the signal’s transmission. In the TDoA method, the anchors must be accurately synchronized since the position estimation is conducted by finding the difference between the time stamps for signal arrival at the anchors [8]. The TWR approach depends on the time that the RF signal requires to travel from the transmitter to the receiver, be processed, and travel back to the transmitter. In this way, synchronization issues are conveniently solved in comparison with the ToA and TDoA methods [9]. By using the TWR method, the need for synchronization between anchors is eliminated. RTT is similar to TWR in terms of calculating the range based on the time it takes for the signal to travel from a transceiver, reach another transceiver, and then be received back.

Utilizing RTT and TWR for range measurement eliminates the need for synchronization in comparison with ToA and TDoA methods, and they are also free of the problems caused by path loss models in RSS-based methods. However, they are not totally trouble-free. ToF-based methods often suffer from a multi-path-effect. This effect is more significant in WiFi RTT compared to UWB TWR, for example, due to limited WiFi bandwidth, and it happens when the signals travel through different paths to reach the receiver instead of traveling directly to the receiver. Consequently, the expected time for the signal to arrive is greatly affected, and the calculated range is based on an inaccurate delay. Finding the accurate delay-based range in an indoor environment with dense multi-path propagation is challenging [7]. Nevertheless, UWB signals have a wide bandwidth that provides the opportunity to filter reflections of the signals to a great extent [10]. With respect to WiFi RTT, not many access points provide a Fine Timing Measurement (FTM) protocol, and not all of those respond to the FTM RTT requests of all the initiators [11]. Therefore, there are limitations in the available devices. Furthermore, there are device-related errors that should be removed from range measurements [12], and yet there are not many available reports using the WiFi RTT method [13]. On the other hand, UWB devices have their own limitations for positioning. Considering the wide bandwidth of these signals, there is interference with other signals and, consequently, restrictions have been imposed on the power gain of UWB signals [9]. This results in lower signal power and ultimately a lower Signal to Noise Ratio (SNR) [14]. In addition, the range measurement accuracy of a device that has an embedded UWB omnidirectional antenna (this type of antenna radiates equal power in all horizontal directions, whereas an isotropic antenna radiates with equal power in all three dimensions [15]) may be highly dependent on the relative orientation of the transmitter and the receiver [16]. Nonetheless, UWB TWR and WiFi RTT are two suitable and well-recognized candidates for use in indoor positioning [17,18,19]. Both of them have benefits that can compensate for the other’s limitations. In addition, RSS values can support ToF-based methods to result in more accurate measurements [7].

The main questions that we seek to answer in this article are: (1) Can we improve the RTT-based ranges achieved with high position-dependent error? (2) Can we achieve better positioning accuracy by fusing UWB and WiFi signals? (3) Which variables and techniques can we use to provide the best fusion algorithm? In this work, considering UWB TWR- and WiFi RTT-based ranging for accurate positioning purposes, we focus on utilizing RSS values as well as a Machine Learning (ML)-based error mitigation method to provide an accurate position calculation. To summarize, the main contributions of this paper are as follows:

- We propose a method with three phases to enhance WiFi-based range measurements, to extract a model for environment observation, and to improve position estimation by way of sensor fusion.
- We investigate the behavior of WiFi devices by analyzing range measurements using data analysis methods. We analyze data collected in an environment with nearly zero multi-path effect (an anechoic chamber), as well as in a real indoor environment with a significant multi-path effect.
- We estimate different ML models to solve the WiFi position-dependent problem that we earlier observed. We take advantage of a supervised learning method to correct the range measurements.
- After correction of WiFi-based range measurements, we utilize a Gaussian Process (GP) as a generic supervised learning method to model the environment. For the implementation of the GP, we utilize random variables for range measurements indexed by time to extract the required variables for sensor fusion.
- In addition to the variables extracted using GP, we take advantage of the effect of RSS on positioning accuracy to improve the proposed range fusion algorithm.
- To evaluate the proposed method, we implement the setups and the algorithm for position estimation in an indoor environment. We consider the separate use of UWB and WiFi as the baseline for our work to show that the range fusion method can provide better results in terms of accuracy.

The remainder of the article is divided into the following sections. Section 2 reviews the ranging and positioning systems utilizing WiFi RTT and UWB TWR. The proposed system model and investigated calibration procedures are presented in Section 3. Section 4 provides the implementations and evaluations, including the analysis of the obtained positioning results. Then, conclusions are drawn in Section 5.

## 2. Overview of WiFi RTT and UWB TWR

WiFi and UWB signals have been previously used in the literature for indoor positioning [20]. In real-world scenarios, there are different challenges that should be addressed to obtain the most benefit from the transmitters and receivers of these signals. In this section, we elaborate on these challenges and the ML-based methods provided in the literature to solve positioning problems.

### 2.1. Utilizing WiFi Signals for Range Measurement

**Figure 1:**
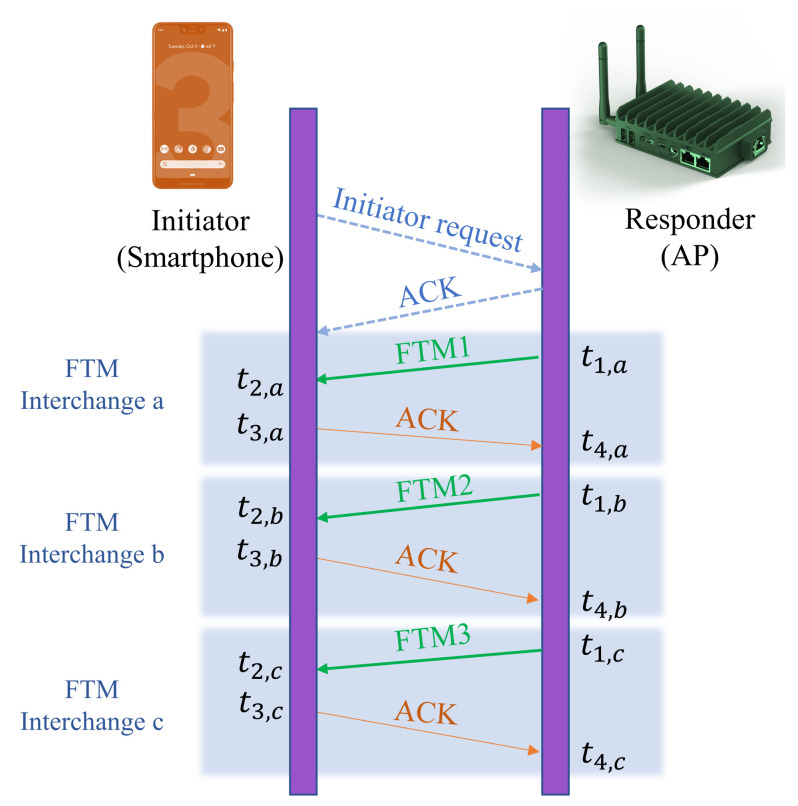
WiFi Fine Timing Measurement (FTM) protocol illustrating one burst with 3 FTM interchanges.

The FTM protocol enables an initiating device to find its distance to a WiFi base station such as an Access Point (AP). The process begins when an initiator (it can be a smartphone) sends an FTM request to an AP. If the AP responds to the request, the process begins and the AP is known as a Responder. A Responder shows its acceptance of the initatior’s message by sending an acknowledgment message (ACK). Then, the Responder sends the initiator an FTM message and waits for the ACK from the initiator. When the initiator sends an ACK to the Responder, the Responder is allowed to send the next message. This procedure is illustrated in Figure 1.

Considering the transmission timestamp of the FTM message and the reception of the ACK in the interchange *k* of a burst, the RTT it takes for a signal to travel from the Responder number *m* to the smartphone and return is estimated:

(1) $$\tau_{RTT,m}=\left(t_{4,k}-t_{1,k}\right)-\left(t_{3,k}-t_{2,k}\right),$$

where *k* is the identity of the interchange in the burst (illustrated by a,b,c in the figure), $t_{4,k}$ is the receiving time of ACK, $t_{1,k}$ is the transmission time of the FTM message, and $t_{3,k}-t_{2,k}$ is the processing time in the smartphone, as illustrated in Figure 1. Several FTM–ACK interchanges occur in one burst. The RTT calculation continues for all the FTM–ACK processes in the burst. Finally, the average value is considered for RTT calculation. Parameters such as the burst duration and the number of bursts are defined during the negotiation phase between the AP and User Equipment (UE) [21].

All the timestamps are transmitted to the smartphone. This allows the UE to calculate the RTT and estimate its position while preserving UE privacy [22]. The RTT calculated in Equation (1) is used to calculate the range between the UE and the AP number *m* as

(2) $$R_{m}=\frac{1}{2}\times \tau_{RTT,m}\times c,$$

where *c* is the speed of light. The UE measures the RTT to all APs in range, which enables the UE to multilaterate its position [3,23]. A significant challenge in positioning using RTT is that the calculated ranges are affected by different types of errors. In [11], these errors in WiFi RTT-based measurements, including position-dependent error, bias, and measurement error, are investigated to increase the accuracy of the calculated ranges. The ranging error of WiFi RTT tested in static positions may be as high as 1.494m, even after bias removal [24]. Similarly, in [25], static test points were used to evaluate the RTT-based ranging calibration. The authors utilized a GP Regression (GPR) to distinguish the Line Of Sight (LOS) from Non-Line Of Sight (NLOS) paths. After LOS identification, nonlinear least-squares fitting was used to enhance the range measurements for LOS distances. The authors implemented their proposed method in an indoor area consisting of two rooms with an area size of 19.5m×5.84m. They were able to achieve a mean positioning error of 2.86m.

Recognizing and removing WiFi device errors and overcoming the errors derived from the multi-path effect are complex problems. ML has been widely used in the literature to address these problems. Dvorecki et al. presented an Artificial Intelligence (AI)-based solution to mitigate the role of the multi-path effect in degrading range accuracy [7]. They utilized Channel Impulse Response (CIR) to observe the first LOS signal path. Using the channel estimation vector, they estimated the delay of the first observed path using a Siamese Artificial Neural Network (ANN). The authors achieved a ranging accuracy of 4m. A general Bayesian filter was presented in [26] to mitigate errors originating from the multi-path effect. The authors integrated RTT range measurement with map information to filter out the RTT-based range errors and provided a position solution with an accuracy of 3m.

A fingerprinting approach was utilized in [15] to localize a smartphone. Fingerprinting is mainly made up of two phases: offline and online. In the offline phase, a large data set of fingerprints for each known position is collected and fed into the system. Later, in the online phase, the test data are compared with the data set, and the collected data are mapped to the corresponding position [27,28,29]. The authors in [15] constructed a distance fingerprint map to train a Convolutional Neural Network (CNN) in the offline phase and optimize the weights and biases in the CNN layers. Then, in the online phase, they fused the optimized model with the range measurement, map information, and accelerometer data used on the UE side. By fusing all the collected data using a particle filter, the authors reported a mean positioning error of 41cm. As another approach, the authors in [30] utilized two neural networks: first, a CNN for correcting the calculated ranges based on RTT measurement and removing the multi-path fading errors, and second, a Recurrent Neural Network (RNN) to estimate the position of the UE by time-series data analysis and fingerprinting. The authors were able to lower the positioning error to 60cm at the cost of a huge data collection procedure for fingerprinting.

Using a combination of WiFi RTT and WiFi RSS was introduced in [23]. A calibration method based on clock modeling was used to improve the accuracy of RTT-based range measurements. Furthermore, a Kalman Filter was utilized to fuse the range measurements based on ToF and RSS to improve robustness. Using the proposed method, the mean positioning error on a set of test points in an indoor environment with an area of $192\;m^{2}$ was 1.435m. The idea of using both WiFi and UWB was presented in [17]. Therein, an indoor environment was divided into different parts, and an UWB base station was used as an intelligent check point in each part. When the user passed one of the UWB stations, it specified that the user was in that part of the building. Then, the results were integrated with the ranges calculated using RSS values and path loss models from visible WiFi base stations. Using this method, a mean error of 2.65m with a standard deviation (STD) of 1.36m was reported.

### 2.2. Range Measurement Using UWB TWR

UWB signals for range measurement have been frequently used in the literature. UWB technology is well-known in the positioning domain to the extent that it is being embedded in flagship smartphones to provide accurate positioning in the coverage area [31]. To find the range between a transmitter and a receiver with UWB signals, one method is time delay measurement. A large bandwidth results in a narrow pulse in time domain, which can be utilized for obtaining accurate delay-based measurements. Considering that a wide pulse captures various reflections from the environment because the signal is scattered by objects such as walls, a narrow pulse has better time resolution and, thus, is more robust against the multi-path effect [32,33]. In UWB systems, the pulse width is in the order of a nanosecond, while the bandwidth is around 500MHz. Consequently, UWB signals filter reflections to a great extent [10]. In addition, these signals propagate through walls and different materials; this makes them a suitable candidate for ranging and positioning purposes [10,34]. However, having a wide bandwidth is not free from side effects. A wide bandwidth increases the interference potential with conventional radio systems; therefore, the power spectral density of a low-pulse-width signal should be limited. Regarding the decision of the Federal Communications Commission (FCC) in the USA and the Harmonized European Standard (EU ETSI EN 302 065), the maximum allowed isotropic radiated power density for unlicensed use of UWB is limited to −41.3dBm/MHz [35]. Signal power affects the SNR and is a significant factor in the precision and accuracy that RF signals can provide [36]. UWB signals with power limitations are not easily distinguishable from the noise floor at the receiver side [37], which results in decreased ranging accuracy due to low SNR [2,38].

UWB TWR-based range measurements do not require synchronization between the transmitter and the receiver as with RTT-based range measurements [39]. UWB TWR is explained in detail in IEEE 802.15.4-2011 [40]. An example of range measurements under static conditions was investigated in [41] by using Pozyx UWB devices. The results showed positioning errors of 1.73m in UWB-based range measurement, and errors of more than one meter for positioning in a dense indoor environment with significant multi-path effects. This error can be increased to 5.919m when the user is moving in a narrow corridor [42]. The effect of static position on reference test points was investigated in [32,42] to analyze ranging capabilities in a favorable environment. Static positioning can also be used in Wireless Sensor Network (WSN) node positioning, where the sensor nodes are fixed [43].

In [44], the authors utilized UWB, Long Term Evolution (LTE) networks, and WiFi anchors. In the proposed method, UE uses UWB and WiFi anchors not located in the same place. LTE is used to cover missing range measurements using RSS Indicator (RSSI) and a propagation model to calculate the range for Weighted Least Square (WLS)-based multilateration. The weights are defined based on range errors for the available anchors in the environment and are updated every 5 s. In good propagation conditions with a low ranging error, the authors achieved a 1.43m mean positioning error. Considering the high errors in estimated reference ranges in dense-multi-path environments, an RSS-based fusion could attain an accurate solution in a shorter time span [45].

In this work, we fuse UWB signals and WiFi signals transmitting from devices fixed in the same location. We utilize RSS measurements to address the multi-path effect and consider the superiority of each signal to compensate for the limitations of the other signal. We utilize GP to take advantage of an environmental observation model in the range fusion without the need for additional equipment or expensive database creation. The advantages and disadvantages of UWB and WiFi are listed in Table 1.

A summary of different methods used in the literature to address the limitations of UWB and WiFi devices is presented in Table 2.

## 3. Proposed Procedure

**Figure 2:**
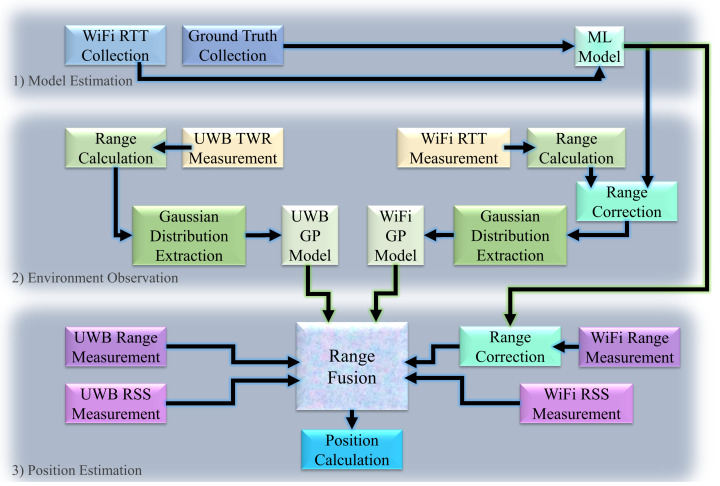
The three phases of the proposed method.

The proposed method has three main phases to minimize the range error and consequently improve positioning accuracy. This section gives a specific description of the proposed phases. The phases are illustrated in Figure 2.

### 3.1. Phase 1: Model Estimation

By investigating WiFi RTT range measurement using the FTM protocol, we observed position-dependent error that was previously observed in the literature [11]. The WiFi device used in this work is the WiFi Indoor Localization Device (WILD) from Compulab company. As mentioned previously, not all smartphones have the ability to send an FTM request. The smartphone we use in this research is a Google Android Phone: Pixel 3.

To elaborate, there are different errors affecting range measurement accuracy. Two of the main types of errors are: (1) the result of NLOS propagation in the test environment and (2) position-dependent error related to the WiFi devices. To model the position-dependent errors of the devices, an investigation should be performed in an area free from reflections and multi-path effects [11]. The multi-path effect has a high potential to cause errors in range values by changing the propagation delay of a signal traveling from a transmitter to a receiver. The presence of the multi-path effect makes it impossible to separate position-dependent errors from those related to the multi-path effect. Therefore, in the first phase, the WiFi RTT-based range measurements are collected inside an anechoic chamber.

#### 3.1.1. Data Collection in an Anechoic Chamber

**Figure 3:**
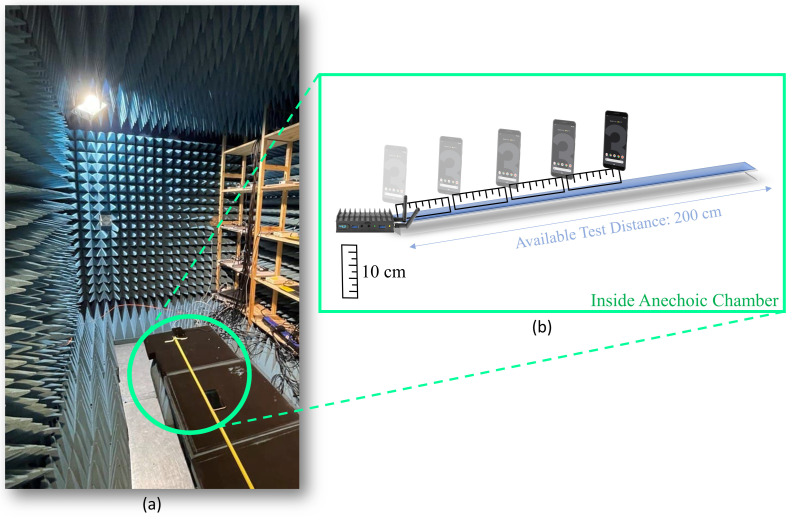
(**a**) Measurement setup in the anechoic chamber. (**b**) Data collection for model estimation.

An anechoic chamber is a room where electromagnetic reflections are absorbed by absorbers installed on the walls, ceiling, and ground. It is also isolated from interfering signals [48]. The required data for model estimation are collected in the chamber of the Department of Computer Science at the University of Helsinki. The chamber and the method for collecting the required data are illustrated in Figure 3.

The anechoic chamber we use has a size of 250cm×240cm×400cm and is constructed using the Rainford EMC Systems modular panel system. This consists of 19mm-thick panels formed from two ‘skins’ of 0.5mm-thick galvanized steel laminated over a core made of wooden material. The whole chamber, including the sidewalls and the ceiling, is covered with 30.5cm-thick, solid, sharp-tip resistive pyramidal foam absorbers (Figure 3a).

The size of the available anechoic chamber allows obtaining the measurements at a maximum distance of two meters. However, considering the research conducted in [11] using WiFi devices from Compulab, we form a hypothesis that a model estimated based on short distances is also valid for longer distances. In the evaluation section, we implicitly test this hypothesis by comparing the positioning accuracy achieved without any error correction to that achieved after performing error correction.

#### 3.1.2. Range Correction using Machine Learning

**Figure 4:**
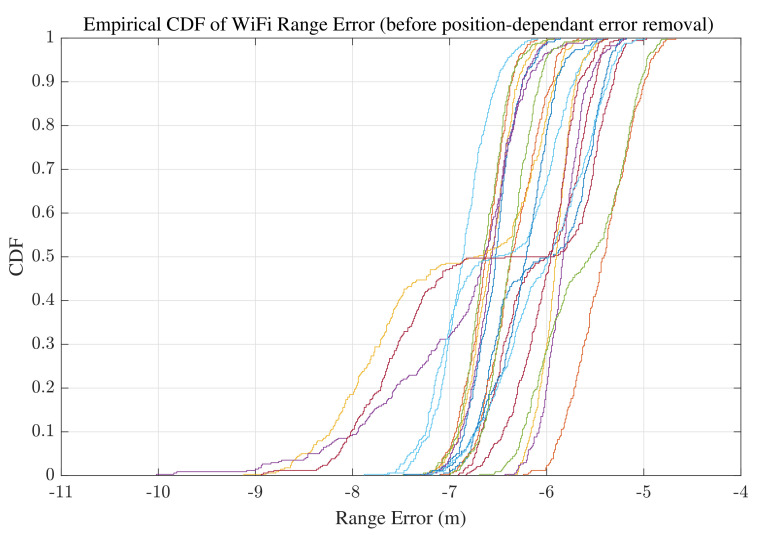
Cummulative Distribution Function (CDF) of range measurement mean error at 21 points in the anechoic chamber.

**Figure 5:**
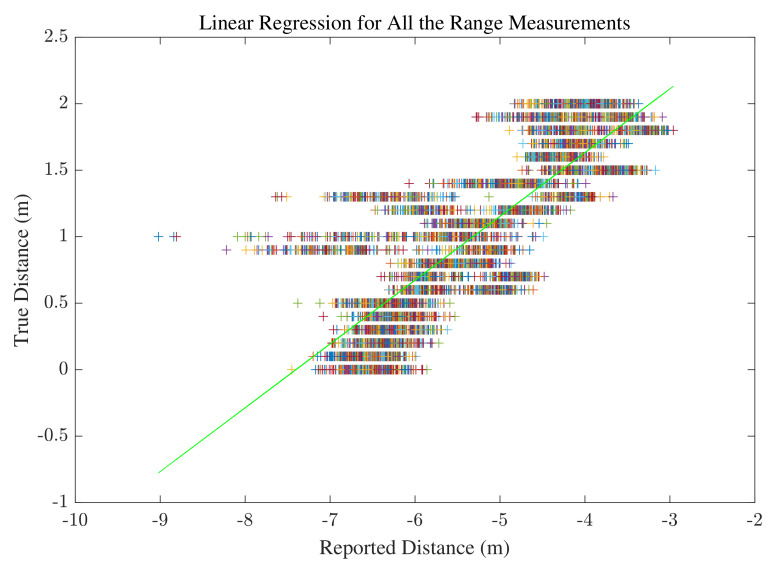
Linear regression applied to all the range measurements.

**Figure 6:**
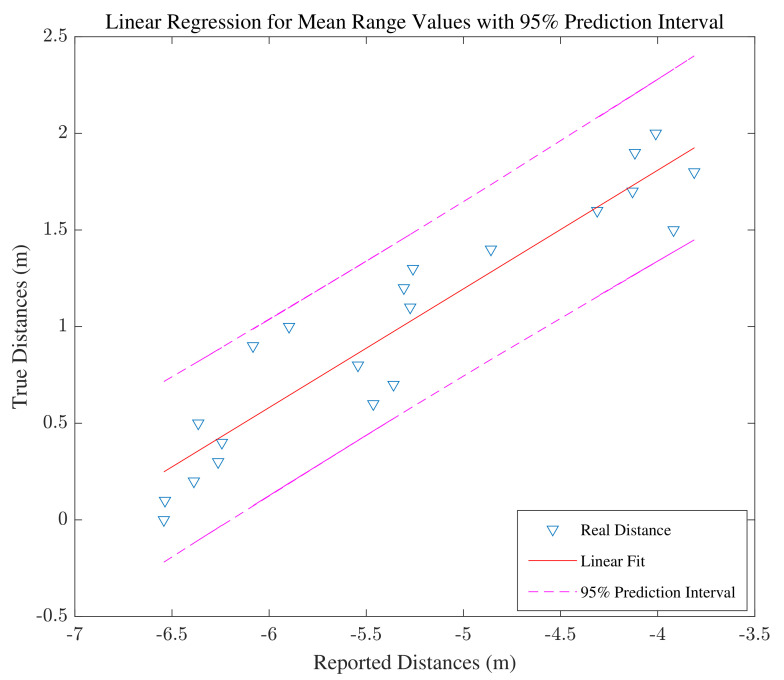
Linear regression for the mean values.

As illustrated in Figure 3b, the smartphone is placed at different distances from the WiFi device in 10cm intervals. The training data are the range measurements collected at different distances, and the labels are the true distances between the mobile phone and the WiFi device. Regarding the known labels, this problem requires a supervised learning method. There are 21 labels and 300 collected ranges at each point. Before applying any range calibration, the error in range measurements in the anechoic chamber is calculated and the Cummulative Distribution Function (CDF) of the mean error at 21 points is illustrated in Figure 4. First, the linear regression method is applied to all the collected data, and the result is illustrated in Figure 5. Linear regression is a supervised ML algorithm frequently addressed in the fundamentals of ML [49,50]. In the linear regression training phase, a data set is used to estimate the model by finding the offset and scale parameter of a line. Then, in the test phase, the new input is provided to the model, and the output is estimated. The linear regression is also applied to the mean value of the collected ranges at each point in the chamber. The resulting regression line is shown in Figure 6.

In order to find the best ML method, other approaches, including Lasso and Ridge regression, Generalized Least Square (GLS), and Support Vector Machine Regression (SVR) are applied, and the ML model is extracted using each method. The reason for considering only regression-based approaches is that the WiFi data set is not huge, and the data are not extremely complicated. Otherwise, neural networks would most likely be a better option, as they are able to cope with more complicated patterns, for example, those found in image processing studies. Lasso regression uses a least absolute shrinkage and selection operator. With respect to shrinkage, this method shrinks the data values toward a central point. This method is explained in detail in [51]. We perform a Lasso fit using a 10-fold cross validation to train and test the model. Cross validation is explained in [50]. Considering the correlation that we observe among the measurements, we also try Ridge regression [51]. This method uses a linear least square as the loss function and takes advantage of L2-norm for regularization. The value of the considered non-zero regularization parameter for Ridge regression in our work is 2. GLS is a generalization of ordinary least squares estimation, and it is used to estimate the unknown parameters of a regression model when there are correlations between the residuals. GLS is further explained in [52]. In this work, we take advantage of feasible GLS, which is an implementable version of GLS. The number of iterations is set to 1. SVR is a supervised learning method that is used for classification problems and can improve the generalization ability of the learning process. We utilize SVR to predict the discrete values. This method tries to find the best fitting line and is further explained in [53]. The kernel scale considered in this work for SVR implementation is 1, the function is linear, the predictor data are not standardized, and a 10-fold cross validation is used. All the utilized methods are supervised learning, and there are 21 labels considered to train the model. The labels are the 21 different distances, as explained previously.

**Figure 7:**
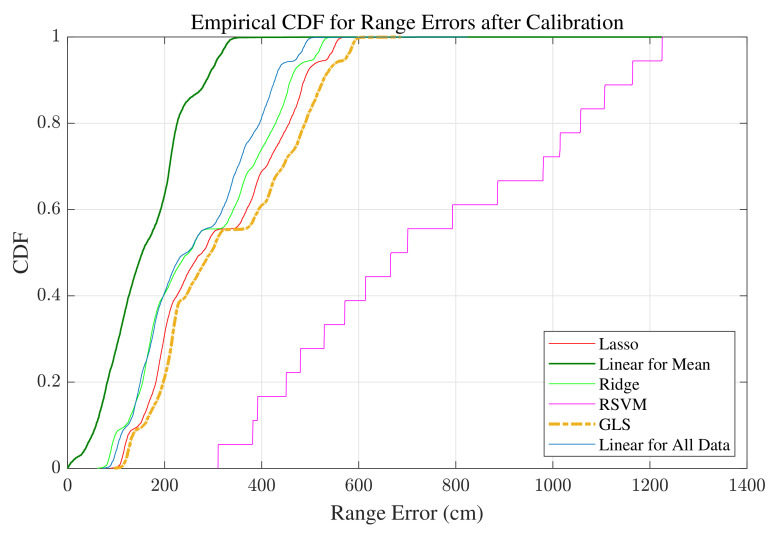
CDF of range errors for the measurements collected in the real environment using the WiFi device.

To find the most efficient model, we apply the different models to correct the RTT measurements in the real test environment. The CDF of the range errors after range correction are illustrated in Figure 7. Based on the findings, the most accurate results are achieved by using the linear regression model made with mean values. Because of highly correlated measurements, it is justifiable that linear regression with averaged values has better accuracy compared to regression with all data [54]. Therefore, the model utilized for the next phases is the model obtained from linear regression with average values. The offset for the estimated model is 4.266, and the scale parameter is 0.614.

### 3.2. Phase 2: Environment Observation

As illustrated in Figure 2, the GP models are used to enable the range fusion method in the third phase. To model the behavior of devices, we extract the Gaussian distributions of the range measurement errors. To extract the range errors, we first apply the ML model to correct the WiFi-based range measurements, as illustrated in Figure 2. By obtaining the parameters of the Gaussian distribution for the range measurements (extracted using the Maximum Likelihood Estimation (MLE)) at different points and updating the posterior based on the new observations, we are able to define the Gaussian process model for device behavior.

The environment model parameters are extracted from measurements taken in the environment of positioning interest. Therefore, the observed environment is indoors, inside the department of computer science at the University of Helsinki. The environment is explained and illustrated in Section 4. The minimum distance considered in modeling the environment is 150cm, and the maximum distance is 1200cm. To model the positioning estimation in the considered environment using WiFi and UWB separately, the parameters for the Gaussian distributions of the position errors are extracted (using MLE). The positions are estimated using the trilateration method (explained in the next subsection), and average range measurements are calculated based on over 300 measurements. Note that the origin of the distribution of errors in range measurements is different from that of position estimations; the positions are estimated using the averaged range values, but the ranging error distribution is calculated by considering all the range measurements. This is also important to mention, considering that WiFi-based ranges in this phase are free from position-dependent error because they are corrected using the ML model, as illustrated in phase 2 in Figure 2. The statistical model of these Gaussian distributions is parametrized by the mean and variance in range errors for UWB $\left(\mu_{r\_UWB},\sigma_{r\_UWB}\right)$ and for WiFi $\left(\mu_{r\_WiFi},\sigma_{r\_WiFi}\right)$, as well as trueness and precision of position calculations using UWB TWR $\left(\mu_{p\_UWB},\sigma_{p\_UWB}\right)$ and WiFi RTT $\left(\mu_{p\_WiFi},\sigma_{p\_WiFi}\right)$. The trueness and precision define the accuracy according to ISO 5725-1, where trueness is the mean and precision is the variance of the error distribution.

To obtain the required values for phase 3, the GPs are modeled based on the product of the Gaussian distributions as a collection of random variables [55]. The parameters describe the behavior of devices in the considered positioning environment, which is enabled with the marginalization property of Gaussian processes [56]. The GP models are used in the third phase for range fusion.

#### Trilateration Method

By having three range values from a UE to three anchors placed in known locations, we are able to locate the UE in a 2D scenario utilizing the trilateration method. This method uses triangle geometry to estimate the position of a mobile object [3,23]. The position of the UE $P_{ue}=\left(x_{ue},y_{ue}\right)$ can be estimated from Equation (3) as:

(3) $$P_{\mathrm{ue}}={\left(Q^{T}Q\right)}^{-1}Q^{T}M,$$

where

(4) $$Q=2\left[\begin{array}{cc} x_{a1}-x_{a2} & y_{a1}-y_{a2}\\ x_{a1}-x_{a3} & y_{a1}-y_{a3} \end{array}\right]$$

and

(5) $$M=\left[\begin{array}{cc} R_{a2}^{2}-R_{a1}^{2}-\left(x_{a2}^{2}-x_{a1}^{2}\right)-\left(y_{a2}^{2}-y_{a1}^{2}\right)\\ \; & \\ R_{a3}^{2}-R_{a1}^{2}-\left(x_{a3}^{2}-x_{a1}^{2}\right)-\left(y_{a3}^{2}-y_{a1}^{2}\right) \end{array}\right].$$

Moreover, $\left(x_{a1},y_{a1}\right)$, $\left(x_{a2},y_{a2}\right)$, and $\left(x_{a3},y_{a3}\right)$ are the known positions of the three anchors, and $R_{a1},R_{a2},$ and $R_{a3}$ are the range measurements from the user to the three anchors.

### 3.3. Phase 3: Position Estimation

**Figure 8:**
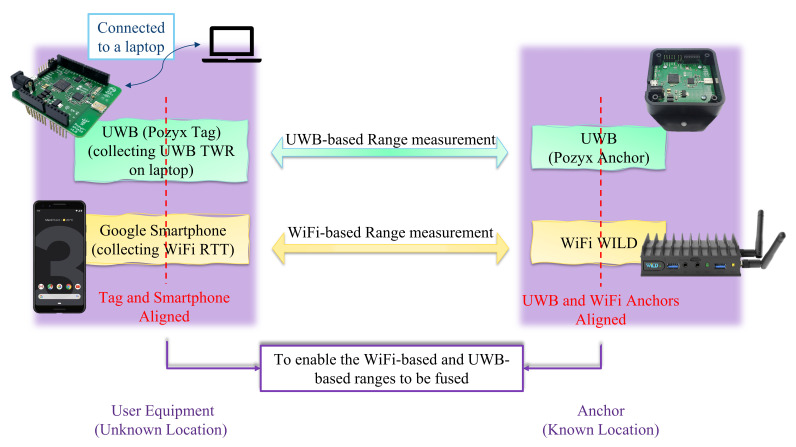
Range measurement using the anchors and User Equipment (UE).

In this phase, the range values are collected by the UE from both the WiFi devices based on RTT and the UWB devices based on TWR measurements. The measurements in this phase are different from those taken in the previous phase. The previous phase is completed once in the indoor environment of research interest. All three devices are used to enable the trilateration method to locate the UE. The UE and one anchor are illustrated in Figure 8.

In the location of each anchor, there is one UWB and one WiFi device. The WiFi RTT measurements are collected with the smartphone, and the UWB TWR measurements are collected with a UWB tag. The UWB tag is connected to a laptop to save the measurements.

#### Range Fusion

To fuse the range measurements collected by the smartphone and the laptop, we consider two main aspects:

1. The GP model parameters extracted from environment observation and device behavior in phase 2.
2. The RSS values corresponding to the range measurements.

The smartphone and the laptop are synchronized using the Network Time Protocol (NTP). Thus, the collected range measurements using WiFi and UWB can be fused together. Otherwise, if the devices are not synchronized, there is a possibility that the ground truth for WiFi-based range measurement might be different from that of UWB at a specific time stamp. In such a case, the range measurement cannot be fused. The update rates for UWB and WiFi devices are different. Let us call them $ur_{UWB}$ and $ur_{WiFi}$, respectively. To find an update rate for the fused measurement, we consider the Greatest Common Divisor (GCD) of the two update rates:

(6) $$ur_{fused}=GCD\left(ur_{UWB},ur_{WiFi}\right).$$

Therefore, in one time epoch, the number of available measurements equals the update rate of the fusion $ur_{fused}$. In the range fusion algorithm, RSS values are utilized to provide robustness against the multi-path effect. Path loss and attenuation result in decreased RSS values [57], and very high RSS, in many environments, indicates a LOS-dominated channel [7]. Thus, the normalized RSS values are the parameters for defining the weights in the fusion method. The higher the RSS value, the higher the weight assigned to the measurement. The following equations are used to calculate the weights for fusing the measurements:

(7) $$w_{1}=\left(\frac{RSS_{\left(UWB,max\right)}}{\sum_{i=1}^{ur_{fused}}{RSS(i)}_{UWB}}\right)\times \frac{\sigma_{p\_UWB}^{2}\times \mu_{p\_WiFi}}{\sigma_{p\_UWB}^{2}+\sigma_{p\_WiFi}^{2}}\times \frac{\sigma_{r\_UWB}^{2}\times \mu_{r\_WiFi}}{\sigma_{r\_UWB}^{2}+\sigma_{r\_WiFi}^{2}},$$

(8) $$w_{2}=\left(\frac{RSS_{\left(WiFi,max\right)}}{\sum_{i=1}^{ur_{fused}}{RSS(i)}_{WiFi}}\right)\times \frac{\sigma_{p\_WiFi}^{2}\times \mu_{p\_UWB}}{\sigma_{p\_UWB}^{2}+\sigma_{p\_WiFi}^{2}}\times \frac{\sigma_{r\_WiFi}^{2}\times \mu_{r\_UWB}}{\sigma_{r\_UWB}^{2}+\sigma_{r\_WiFi}^{2}},$$

where $RSS_{\left(UWB,max\right)}$ and $RSS_{\left(WiFi,max\right)}$ are the maximum RSS values in every $ur_{fused}$ measurement. RSS values are in watts. $\sum_{i=1}^{ur_{fused}}{RSS(i)}_{UWB}$ and $\sum_{i=1}^{ur_{fused}}{RSS(i)}_{WiFi}$ are the sum of RSS values for all the $ur_{fused}$ measurements from UWB and WiFi devices, respectively. Larger RSS values imply a stronger signal and result in higher $w_{1}$ and $w_{2}$. In the second and third fraction of Equations (7) and (8), the parameters of GP in environment observation and device behavior in the environment are applied.

The coefficients to fuse the ranges are finally defined as

(9) $$Coeff_{UWB}=\frac{w_{1}}{w_{1}+w_{2}}\;\mathrm{and}$$

(10) $$Coeff_{WiFi}=\frac{w_{2}}{w_{1}+w_{2}}.$$

Before the fusion, the WiFi RTT-based range measurements are calibrated using the ML model estimated in the first phase (Figure 2). Thus, the final fused range value is defined as

(11) $$Range_{fused}=Coeff_{WiFi}\times R_{\left(Wifi,max\right)}+Coeff_{UWB}\times R_{\left(UWB,max\right)},$$

where $R_{\left(Wifi,max\right)}$ represents the corresponding corrected range to the maximum RSS in every $ur_{fused}$ range measurement based on WiFi RTT, and $R_{\left(UWB,max\right)}$ represents the corresponding range to the maximum RSS in every $ur_{fused}$ range measurement based on UWB TWR.

To clarify the fusion procedure, the algorithm is explained in Algorithm 1.
**Algorithm 1** Range Fusion Method.    **Input:** The ML model estimated in the model estimation phase;    The behavior of the devices (mean and variance of the error in range measurement;     The trueness and precision (generated in the environment observation phase).    **Output:** Fused range values.1:**for** each epoch **do**2:    Find and normalize the maximum RSS value of UWB signals in current epoch;3:    Save the UWB-based range corresponding to the maximum RSS as $R_{\left(UWB,max\right)}$;4:    Calculate w1 using Equation (7);5:    Find and normalize the maximum RSS value of WiFi signals in current epoch;6:    Correct the WiFi RTT-based ranges using the ML model;7:    Save the corrected WiFi-based range corresponding to the maximum RSS as $R_{\left(WiFi,max\right)}$;8:    Calculate w2 using Equation (8);9:    Calculate UWB weight using Equation (9) and save as $Coeff_{UWB}$;10:    Calculate WiFi weight using Equation (10) and save as $Coeff_{WiFi}$;11:    Fuse $R_{\left(UWB,max\right)}$ and $R_{\left(WiFi,max\right)}$ using their correspounding weights;12:**end for**

## 4. Implementations and Evaluations

**Figure 9:**
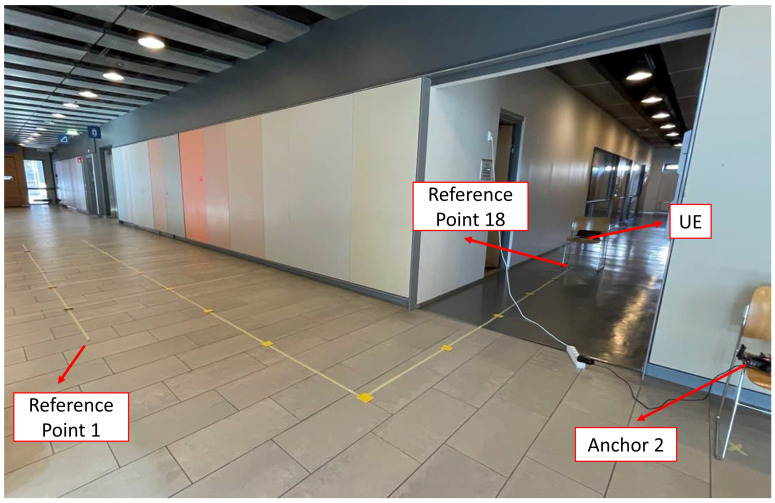
Reference points for evaluating the results inside the hall and the corridor.

**Figure 10:**
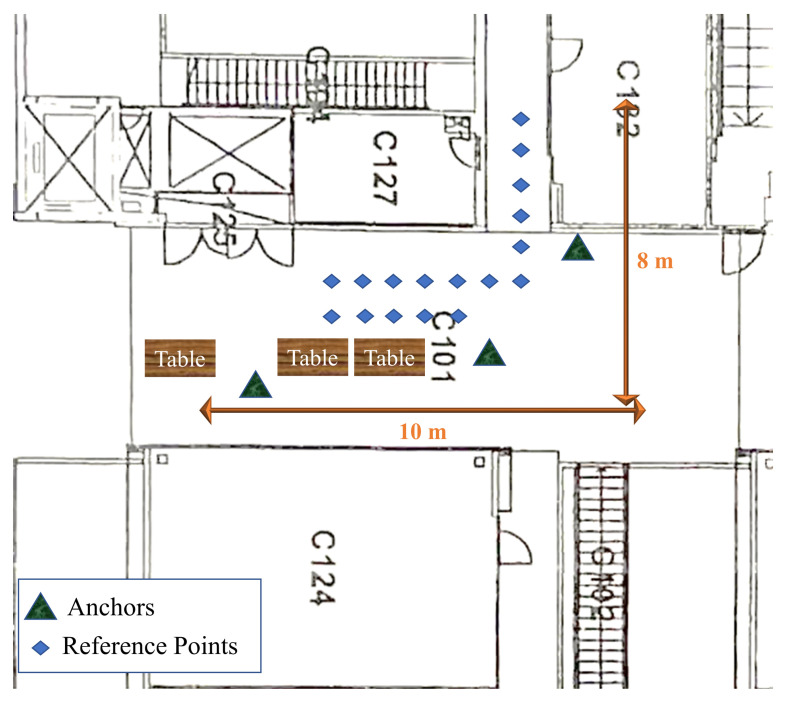
Floor plan of the area of measurement campaign.

**Figure 11:**
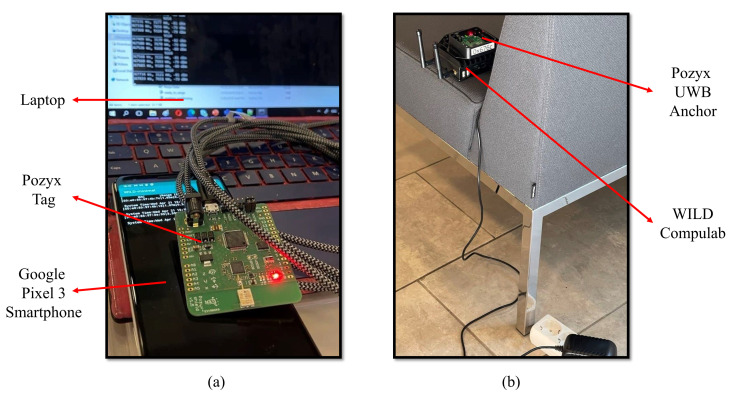
(**a**) The devices used for data collection on the UE side. (**b**) Configuration of devices at the location of one anchor.

Static positioning is widely utilized to evaluate the enhancement of range measurements [23,24,25,32,42]. We evaluate our work by positioning using trilateration of reference points. The test area is inside the department of Computer Science at the University of Helsinki. The reference points are marked with yellow signs, as illustrated in Figure 9. The yellow marks are located using a measurement tape which has an accuracy of one millimeter. However, the accuracy of placement of the devices is one centimeter, considering the placement of the antenna chip inside the mobile phone and the UWB and WiFi devices. In addition, the floor plan of the area for the measurement is illustrated in Figure 10. There are three anchors placed in the hall, and the UE moves on the reference points inside the hall and the corridor. One of the anchors and the UE are shown in Figure 11.

The purpose of putting a UWB device on the smartphone, as illustrated in Figure 11, was to provide a similar setup to smartphones that utilize an embedded UWB antenna. The placement of the antenna should be performed with caution, as it can affect the results and it might distort the omnidirectional characteristic of the UWB antenna.

In the following subsections, we first provide the results obtained using UWB and WiFi devices separately. UWB and WiFi have been used separately in the literature, and we consider this as the baseline for comparison with the proposed method.

### 4.1. Positioning Based on WiFi RTT

**Figure 12:**
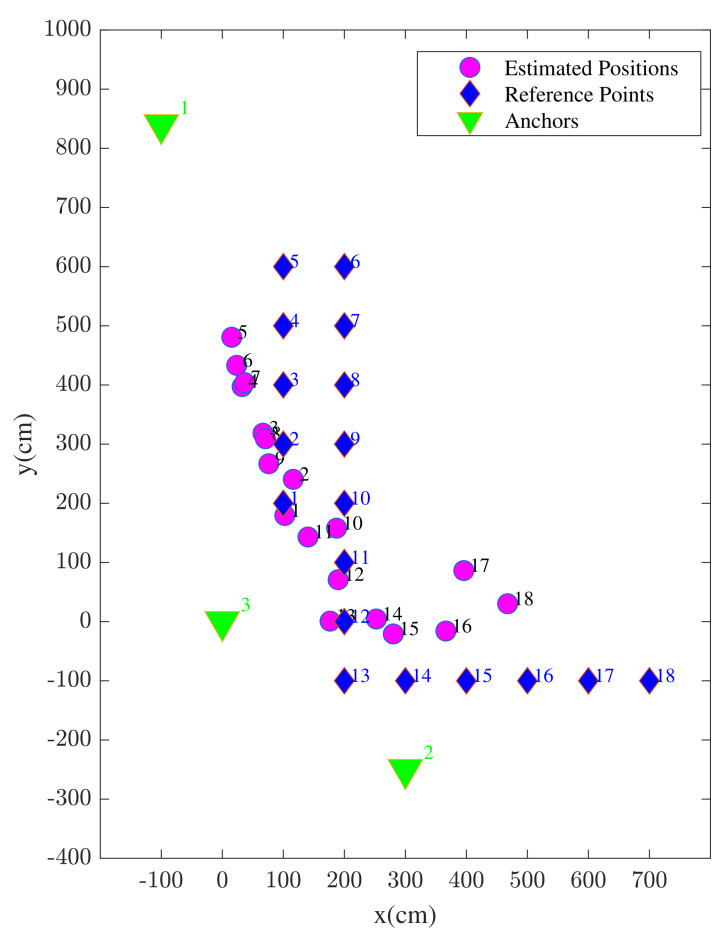
UE positions estimated using standalone WiFi devices.

The position solutions estimated using enhanced WiFi range measurements are illustrated in Figure 12. In the figure, both the reference points and the corresponding position estimates are shown associated with the index of each reference point. It is shown that, most likely due to considered anchor geometry, the estimation accuracy is better towards the center of the study area compared to the edges.

**Figure 13:**
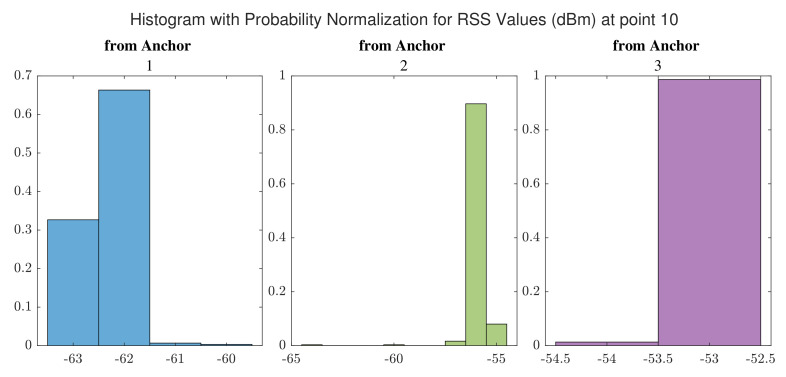
Distribution of Received Signal Strength (RSS) values received from WiFi anchors at point 10.

**Figure 14:**
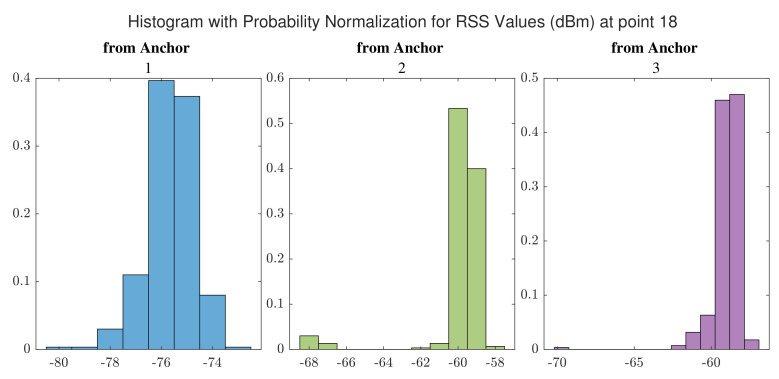
Distribution of RSS values received from WiFi anchors at point 18.

The distributions of RSS values for signals received from WiFi anchors at point 10 and at point 18 are shown in Figure 13 and Figure 14, respectively. 300 measurements were taken into account to prepare these histograms. Based on Figure 13 and Figure 14, it can be observed that the RSS values for a point inside a corridor have lower levels than those for a point placed in the hall where there are available LOS paths from the anchors.

### 4.2. Positioning Based on UWB TWR

**Figure 15:**
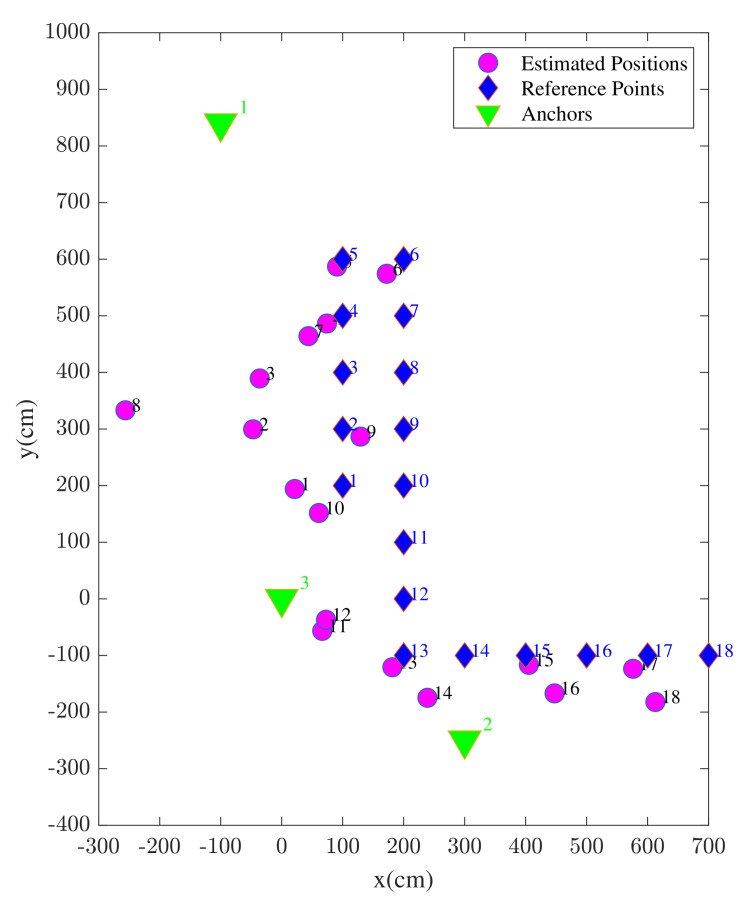
UE positions estimated using standalone Ultra Wide Band (UWB) devices.

The position solutions estimated using UWB TWR-based ranges are illustrated in Figure 15. Contrary to the previously shown WiFi RTT scenario, the position estimates are more widely distributed across the study area. In addition, a clear outlier estimate can be seen regarding reference point number 8.

**Figure 16:**
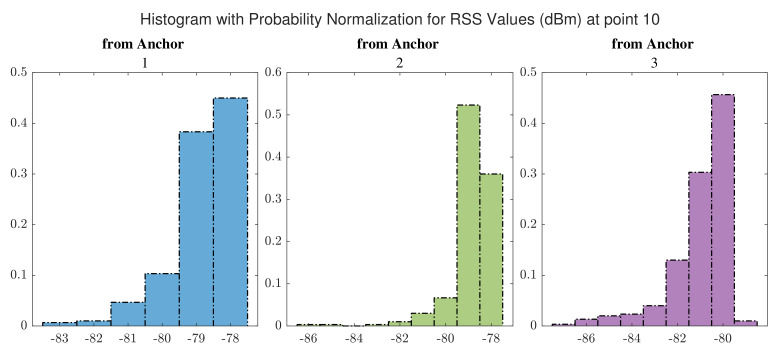
Distribution of RSS values received from UWB anchors at point 10.

**Figure 17:**
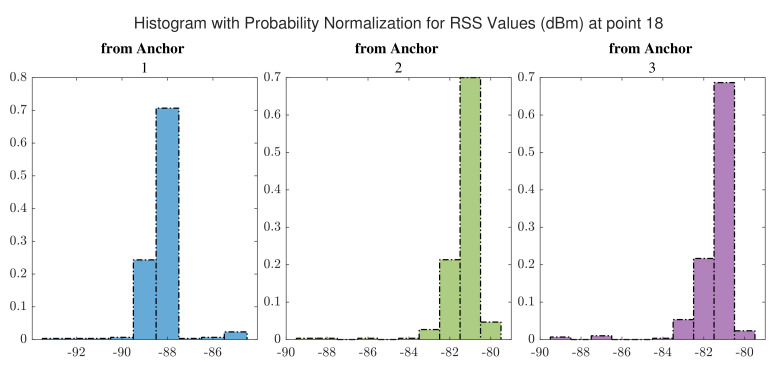
Distribution of RSS values received from UWB anchors at point 18.

The distributions of RSS values for signals received from UWB anchors at point 10 and at point 18 are shown in Figure 16 and Figure 17, respectively. By comparing with Figure 13 and Figure 14, we realize that a point inside the hall that offers LOS paths for signals receives higher signal power compared to the points inside the corridor, especially those signals received from the furthest anchor (anchor 1).

### 4.3. Positioning with Fused Range Measurements Using the Proposed Method

**Figure 18:**
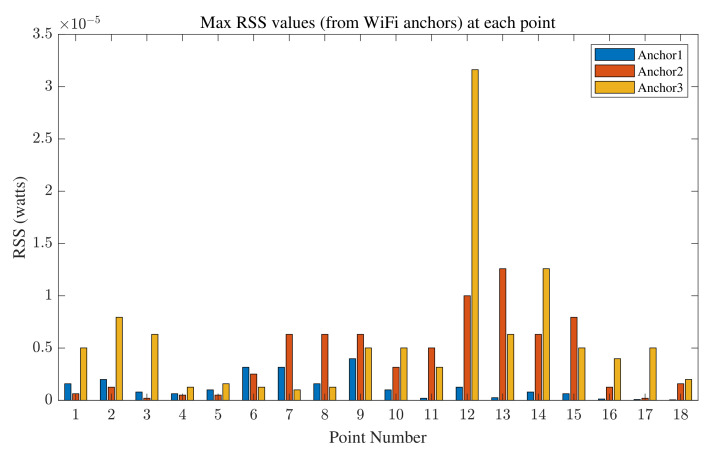
Maximum RSS values in one epoch at each reference point for signals received from three WiFi anchors.

**Figure 19:**
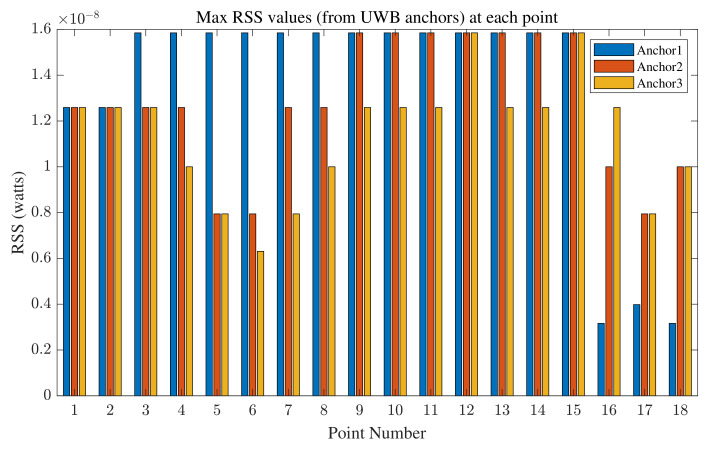
Maximum RSS values in one epoch at each reference point for signals received from three UWB anchors.

The maximum RSS values are utilized in the proposed range fusion algorithm. The corresponding values received from WiFi devices in one epoch at all the reference points are shown in Figure 18, and those received from UWB anchors are shown in Figure 19. By comparing Figure 18 and Figure 19, it is observed that the RSS values for UWB signals are lower than those for WiFi signals as a result of regulations on UWB that require it to have lower power gain. Furthermore, the maximum RSS values at points inside the corridor in the test area (mainly points 16, 17, and 18) display the smallest RSS values from the anchors. It is also observed that the RSS values for points inside the corridor are the smallest for those signals being received from anchor 1, which is the farthest anchor.

**Figure 20:**
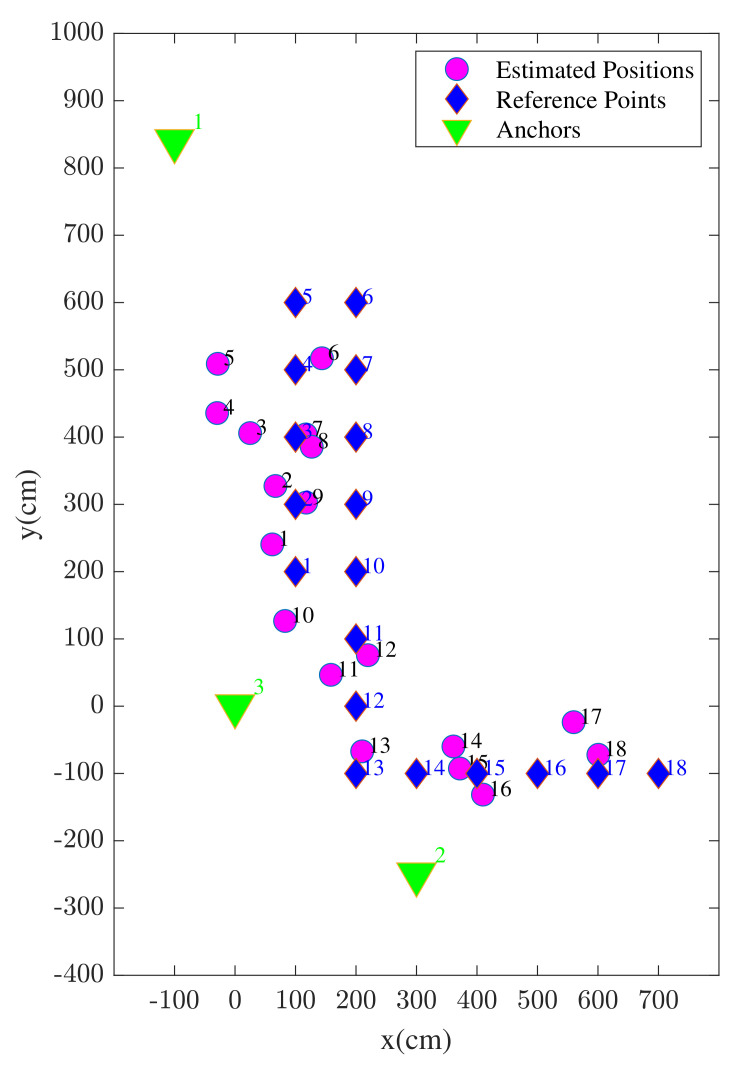
UE positions estimated using the proposed method.

The position solutions estimated using the proposed method are illustrated in Figure 20. By comparing the results of the proposed hybrid approach in Figure 20 with Figure 15 (UWB) and Figure 12 (WiFi), a considerable improvement in the positioning accuracy is visible. In addition to the improved average positioning accuracy, there are no clear observable outliers seen in the results.

### 4.4. Comparison

**Figure 21:**
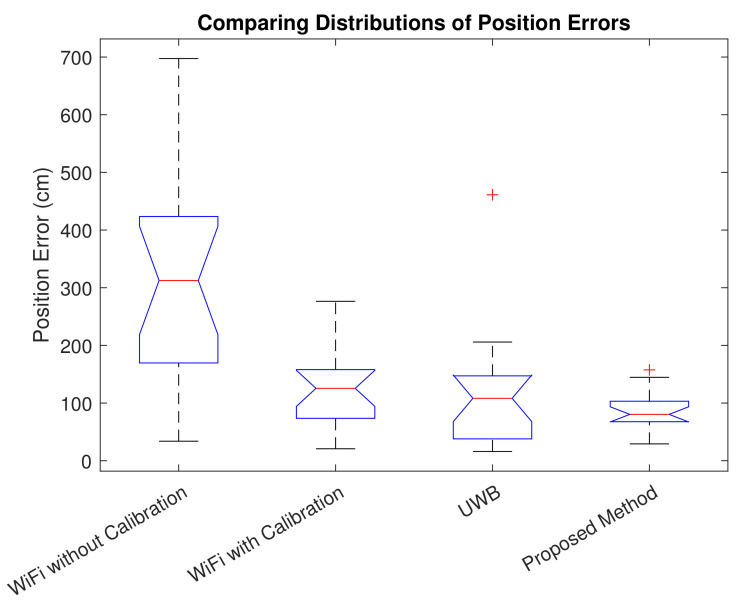
Box plots showing positioning errors using different methods. The proposed hybrid method provides the best accuracy with the smallest error variance.

To evaluate and analyze the results numerically, the positioning errors when using calibrated and non-calibrated WiFi devices, UWB devices, and the proposed method are illustrated using box plots in Figure 21. In the notched box plots in Figure 21, the red lines represent the median value, the length of the dashed lines represents the whiskers, the gray horizontal upper line is the non-outlier maximum, the lower one is the non-outlier minimum, the upper end of the box is the 75th percentile, the lower end of the box is the 25th percentile, the notch represents the 95% confidence interval of the median, and the red plus signs show the outliers. The box plots are not influenced by outliers and visualize the distribution of data in a stable way. The proposed method has the lowest median in Figure 21 and the least variance in comparison with other methods. WiFi with calibration also outperforms WiFi without calibration to a good extent. The results show that the proposed error correction approach is able to considerably improve positioning accuracy.

Table 3 shows the root-mean-square-error (RMSE), mean error, and maximum error in positioning using different methods. Compared to positioning using UWB or WiFi anchors alone, the proposed method reduces the mean error, and the RMSE is improved. Considering that RMSE represents positioning stability [25], the proposed method provides better stability in comparison with other methods.

To demonstrate the significance of the proposed variable-weighted method, we also evaluate performance using simple averaging-based fusion of the UWB- and WiFi-based range measurements. With the simple averaging method, the positioning accuracy is degraded to a mean value of 95cm, an RMSE of 106cm, a maximum error of 188cm, and an STD of 47cm. As considered in Equation (11), we utilize the corresponding range to the maximum RSS value. To demonstrate the effectiveness of this method, we compare our results to methods using the mean range value or the minimum range value instead. The results are listed in Table 4.

**Figure 22:**
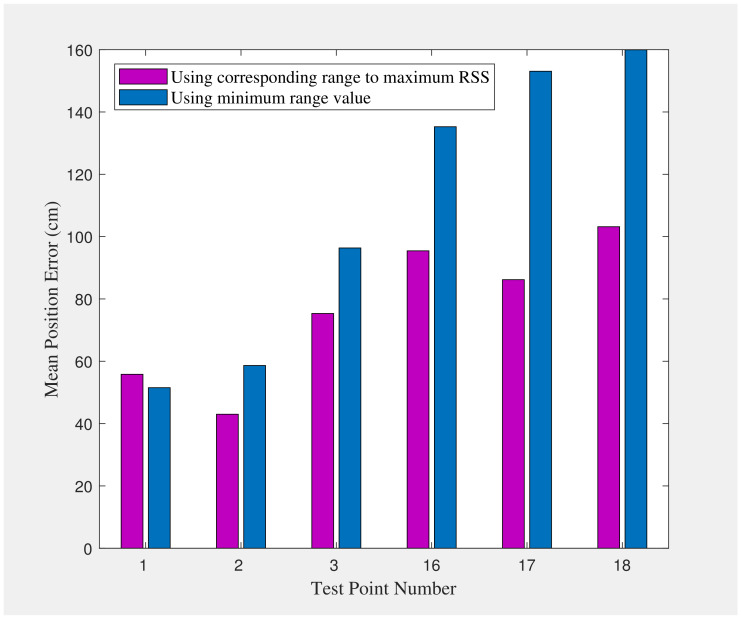
Position error at three Line Of Sight (LOS) and three Non-Line Of Sight (NLOS) points using several range selection methods.

To investigate the impact of NLOS conditions on positioning accuracy, we consider three points in the corridor (points number 16, 17, and 18) as the points in NLOS condition and three other points (points number 1, 2, and 3) as the points in LOS condition. The results are illustrated in Figure 22.

We can observe that the NLOS points have higher error values in their position estimates than the LOS points, which shows the impact of NLOS conditions on positioning accuracy. We can also observe that LOS points have higher RSS values than NLOS points, as illustrated in Figure 18 and Figure 19. These figures, together with Figure 22, show how RSS values affect positioning accuracy and demonstrate our motivation for considering RSS values in the proposed fusion method. The proposed method outperforms using minimum range values. It is observable that the proposed method provides a consistent solution, and the results do not vary highly (which results in minimum variance, as listed in Table 4 and shown in Figure 21).

### 4.5. Issues and Costs of Implementation

The UWB devices used in this research are Pozyx Creator tags and anchors. We used the Python library provided by Pozyx to write our own script for data collection. We used Pozyx software for updating the firmware of the devices and changing parameter values such as update rate. The proper driver was also installed for the Pozyx tag to be connected to a laptop. We faced no issue in the implementation of the Pozyx devices. The most important challenge was to define the power gain in a way that agreed with the regulations, as previously explained. A Pozyx Creator kit costs approximately EUR 1050. It contains five Pozyx anchors, four Pozyx tags, three power banks, and five power adapters. However, we only used three Pozyx anchors (powered by the power banks) and one Pozyx tag for the user, considering the goal of this research. The WiFi devices utilized in this research are WILD Compulab. We developed an Android application and installed it on the Google Android phone. This application collects and saves the RTT measurements. The biggest issues with the WILD devices were the position-dependent errors, which we solved to a good extent in the first phase of our proposed method. Each WILD device cost approximately USD 265 (excl. VAT) at the time of buying the devices. The Google Pixel 3 phone cost approximately EUR 700 at the time of purchase.

## 5. Discussion and Conclusions

A method of enhanced range measurement for indoor positioning purposes is introduced in this work. The proposed method considers the advantages and disadvantages of two well-known RF signals for indoor positioning: UWB and WiFi. The proposed method addresses the shortcomings of each by taking advantage of the strengths of the other. In the first phase of the proposed method, as illustrated in Figure 2, an ML method is estimated to improve range measurements calculated using WiFi RTT. In the second phase, the environment is observed, and the devices’ behavior in the environment is investigated to model GPs. In the third phase, the estimated ML model, GPs, and RSS values are used to provide a robust, accurate, and stable sensor fusion method. The RSS values are examined in Figure 18 and Figure 19, and they are shown to greatly impact the accuracy of range measurement. By analyzing RSS values, we are able to define weights for the range measurements and improve delay-based range calculations.

The results summarized in Table 3 prove that utilizing the proposed method improves positioning accuracy. Considering that there is no need for additional equipment or laborious database creation in the position estimation phase, the proposed method results in lower error values in comparison with its counterparts. To the best of our knowledge, this method is the first method in the literature proposing base stations created using UWB and WiFi anchors and a suitable algorithm to fuse their data.

As a focus for future work, considering that UWB antennas are omnidirectional, we are interested in investigating the effect of antenna direction on ranging accuracy. Furthermore, we are interested in applying our method in a scenario with appropriate tracking algorithms for a navigation application.

## Figures and Tables

**Table 1:** The advantages and disadvantages of utilizing UWB and WiFi for positioning.

Signal and Technology	Advantages	Disadvantages
WiFi RTT	Low Cost [44], Better Energy Performance [46]	Device Dependent Errors [11], Not Widely Reported [13]
UWB TWR	High Precision [32], Robustness Against Multi-path [47]	High Cost [31], Not Strong Transmission in All Directions [16], Power Limitations and Restrictions [9]

**Table 2:** Proposed methods in the literature to overcome UWB and WiFi limitations for positioning purposes.

Reference	Signal and Technology	Method	Mean Positioning Error
[25]	WiFi RTT	GPR and LOS/NLOS detection	2.86m
[30]	WiFi RTT	Fingerprinting, scanning environment, and ANN	60cm
[23]	WiFi RTT and RSS	Kalman Filter-based fusion of RTT and RSS	1.435m
[7]	WiFi CIR and RTT	Finding first LOS path using ANN	4m (Ranging)
[15]	WiFi RTT and accelerometer	CNN for fingerprinting, map information, and particle filter for data fusion in a low multi-path environment	41cm
[17]	UWB sensor, WiFi RTT, and RSS	Intelligent UWB checkpoints and fusion of WiFi RSS-, RTT-based distances	2.65m
[44]	Using WiFi and UWB anchors and LTE	Weighting different base stations	1.43m
The proposed method	WiFi RTT and UWB TWR	Device calibration and RSS-based fusion	87cm

**Table 3:** Estimated position errors with respect to each method.

Algorithm	WiFi without Calibration	WiFi with Calibration	UWB	Proposed Method
RMSE (cm)	362	152	156	94
Mean Error (cm)	318	134	120	87
Maximum Error (cm)	697	276	461	158

**Table 4:** Estimated position errors with respect to range utilization method.

Algorithm	Minimum Range	Average Range	Corresponding Range to Maximum RSS (Proposed)
RMSE (cm)	116	135	94
STD (cm)	52	105	37
Mean Error (cm)	105	88	87
Maximum Error (cm)	220	448	158

## Data Availability

Not applicable.
